# Gastric ischemia as an under-reported cause of death in older people

**DOI:** 10.1007/s12024-024-00840-5

**Published:** 2024-05-28

**Authors:** Maria Paola Bonasoni, Filippo Pirani, Federica Grimaldi, Paolo Fais, Arianna Giorgetti, Susi Pelotti

**Affiliations:** 1 Unit of Legal Medicine, Department of Medical and Surgical Sciences, University of Bologna, Via Irnerio 49, 40126 Bologna, Italy; 2 Pathology Unit, Azienda USL-IRCCS di Reggio Emilia, Via Amendola 2, 42122 Reggio Emilia, Italy

**Keywords:** Forensic pathology, Bowel ischemia, Systemic hypoperfusion, Nonocclusive ischemia

## Abstract

Acute gastric ischemia is a rare condition due to the rich vascular supply of the stomach. Here we present a case of fatal gastric ischemia associated with bowel ischemia, only diagnosed at autopsy, which was requested for the suspicion of medical liability. A complete post-mortem examination was conducted, along with a macroscopic analysis of the superior mesenteric artery and detailed histological analyses. Past clinical data was also reviewed. The macroscopic blackish discoloration of the stomach and the bowel, coupled with the presence of neutrophils in the mucosa and submucosal non-occlusive thrombi, were consistent with gastric and bowel ischemia, despite the presence of confounding putrefactive changes. The unique aspect of this case resides in the ante-mortem peculiar vascularization of the stomach, supplied by small collateral vessels. No mechanical occlusion was identified, and the cause of the ischemia was deemed as non-occlusive, likely due to systemic hypoperfusion. The analysis of clinical data and documentation of associated comorbidities are strongly recommended, especially when a rare cause of death is suspected. With the aging population, especially among women, and the prevalence of risk factors, the forensic pathologist could increasingly encounter rare cases of gastric ischemia.

## Introduction

Acute mesenteric ischemia (AMI) represents a significant cause of morbidity and mortality of older people, although it is not listed among the most common causes of death in this subpopulation [1, 2]. Indeed, its identification is challenging in the clinical setting and the diagnosis is often established only after a post-mortem examination [3]. For several years now forensic pathologists have been warned that, with the general aging of the population, especially in Western countries, bowel ischemia could be increasingly encountered in the forensic practice [3].

The population over the age of 65 is expected to reach 30% by the year 2050 [4], and women over the age of 85 will increase to 40 million in the European Region by 2050 [5]. Women have a longer life expectancy but are also more vulnerable to ill-death [5]. Despite the decline in performed autopsies, post-mortem examination might help the diagnosis of diseases in older people and ensure a higher quality of gender-sensitive medical care [6].

Gastric ischemia represents an even more uncommon condition, as the stomach is highly supplied with blood from the celiac trunk and its divisions, which comprise the left gastric, splenic, and common hepatic artery [7, 8]. The left gastric artery supplies the proximal lesser curvature. Small terminal arteries branch off from the splenic artery to irrorate the gastric fundus. The left gastroepiploic artery also originates from the splenic artery and surrounds the greater curvature. Lastly, the common hepatic artery branches off the right gastric artery, that loops around the antrum and the distal part of the lesser curvature, and the gastroduodenal artery, which distally becomes the right gastroepiploic artery, the vascular supply for the antrum and distal greater curvature [9].

Like AMI, gastric ischemia could be acute or chronic [7] and is typically caused by general or local impairment in blood perfusion [9, 10]. Only a handful of cases have been described in the literature, all reported in a clinical setting or shortly after surgery [9, 11–22]. Combined mesenteric and gastric ischemia were reported due to simultaneous occlusion of the celiac axis and superior mesenteric artery (SMA) [23, 24].

Endoscopy and computed tomography represent the gold standards to establish a clinical diagnosis [7, 9, 25], but the disease is frequently under-recognized and under-reported [9] and might give rise to medical liability, similarly to AMI [26]. Nevertheless, due to the rich vascular supply of the stomach and the low prevalence, pathologists might not be aware of the disease, especially when confounding putrefactive changes occur.

Here we present a case of fatal gastric ischemia associated with bowel ischemia, only diagnosed at autopsy, after the patient was discharged from the hospital with mild gastrointestinal symptoms.

## Case description

An 84-year-old woman presented to the Emergency Department with abdominal pain, vomiting, and diarrhea persisting for 4 h. On physical examination her blood pressure was 160/80 mmHg and she had abdominal tenderness, but no signs of peritoneal irritation. The ultrasound examination showed distended and hyperperistaltic intestinal loops. Laboratory results revealed high levels of C reactive protein (9.65 mg/l), elevated white blood cells count (14,030 /μl), and anemia (10.4 g/dl). Analgesic therapy (paracetamol) was administered and the patient was discharged two hours after admission with a diagnosis of enteritis. Six hours later, she was found dead at home.

The deceased had a history of intramural hematoma of the aortic arch and abdominal aortic aneurysm, both of which were surgically treated with aortic endoprosthesis eleven years prior. As a result, the medical examiner suspected a misdiagnosed prosthetic rupture. The Public Prosecutor appointed the forensic pathologist to identify the cause of death and to investigate the potential medical liability.

### Autopsy findings

The post-mortem examination, performed 7 days later, showed the absence of any traumatic injury. External examination revealed a few signs of putrefaction, and specifically a marked greenish discoloration of the abdominal wall in the upper left quadrant, which corresponded to a greenish discoloration of the intercostal spaces and of the abdominal fat.

Pleural adhesions in the left pleural cavity, with no effusion, and pericardial adhesions were seen. The lungs and heart weight (1000 g and 340 g) were in the normal range. The coronary arteries were both calcified for approximately 2 cm from the origin, but were patent with no hemodynamically significant stenosis.

**Fig. 1:**
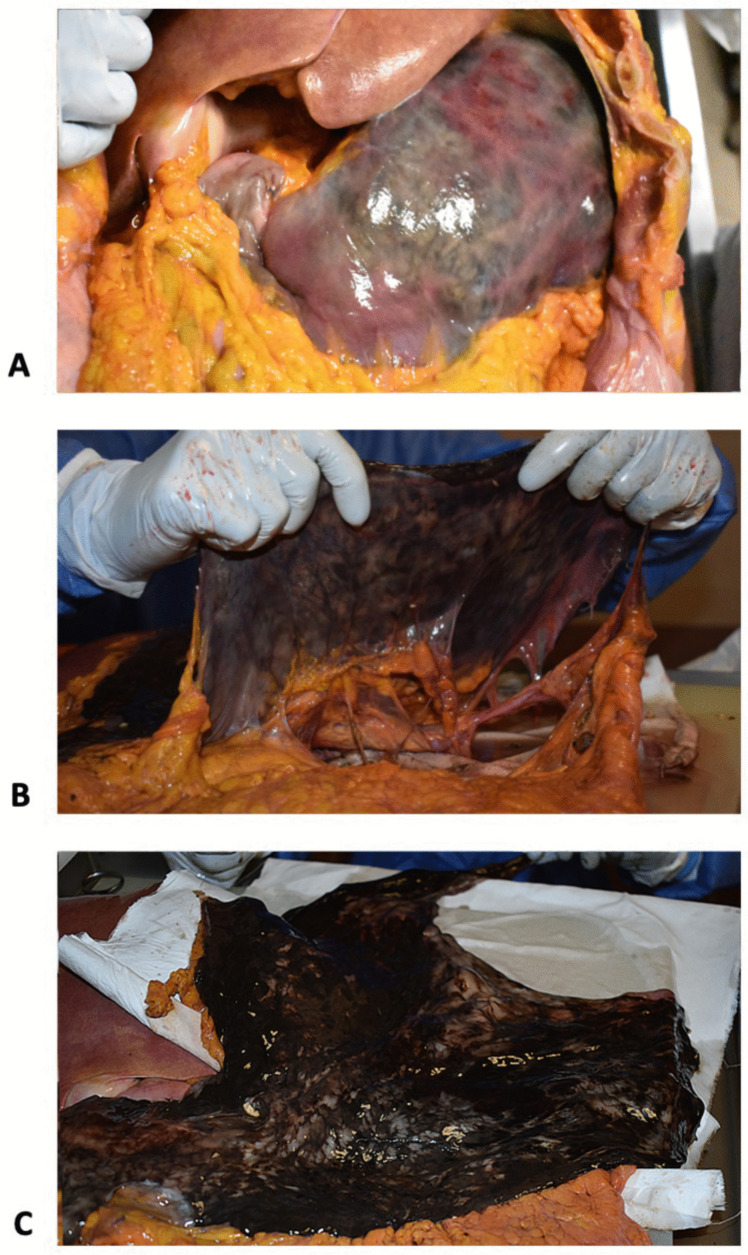
Gastric ischemia/infarction: the stomach showed a diffuse blackish appearance, from the serosal surface (**a**, **b**) to the mucosa (**c**), and a severe thinning of the wall

The endovascular aortic prosthesis was confirmed to be properly positioned, extending from the thoracic aorta to the bifurcation of the iliac arteries, with no signs of perforation. The prosthesis showed an occluded celiac trunk and multiple pervious stents in the SMA and in both renal arteries. Severe atherosclerosis was detected in the aorta, supra-aortic vessels, basilar artery and iliac arteries. The stomach was thinned and characterized by a blackish discoloration, from the mucosa to the serosal surface, which extended to the first part of the bowel (Fig. 1).

**Fig. 2:**
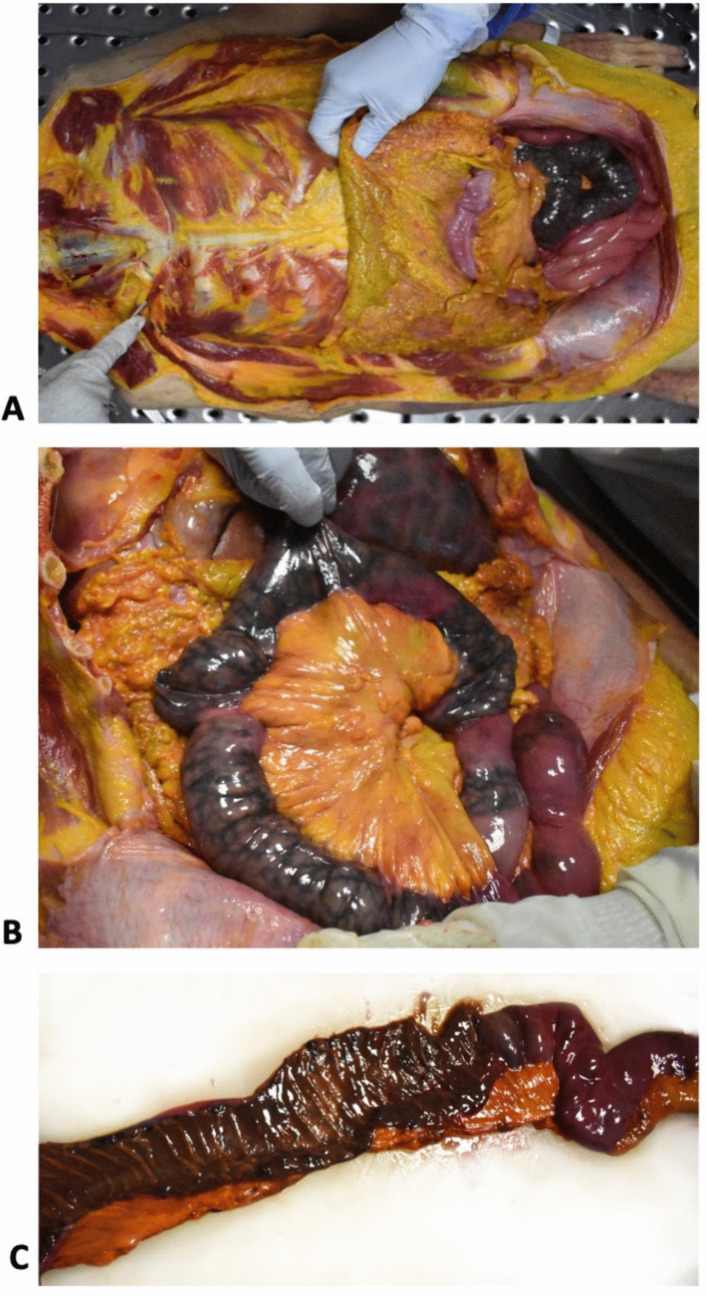
Intestinal ischemia/infarct: the duodenojejunal flexure and the first tract of the jejunum starting from the ligament of Treitz presented a sharp, segmental, and blackish appearance involving the whole wall, from the serosa (**a**, **b**) to the mucosa (**c**)

The duodenojejunal flexure, and the first tract of the jejunum, for a 16 cm length starting from the Treitz ligament, also displayed a segmental and sharp blackish discoloration, consistent with intestinal ischemia/infarction (Fig. 2).

No other significant pathological findings were observed, particularly the other small intestinal loops and the large bowel appeared normal. The SMA and a portion of intestine, at the junction between the black discoloration and the normal tissue, were collected during autopsy and fixed in formalin. The collateral vessels of the celiac trunk were not sampled, due to difficult visualization. The liver (weight: 1460) was unremarkable. Some gallstones were identified in the gallbladder, with obstruction of the bile ducts. The adrenal glands showed no abnormalities, while the kidneys showed irregular surface with some cysts, cortical thinning and blurred cortico-medullary junction.

Samples of tissues were collected for histology.

### Clinical data requested after the autopsy

Additional health data was requested after the post-mortem examination, to assess whether the patient suffered from additional diseases or had attended the Emergency Department in the previous months. From the acquired CTs, performed for several years, a significant reduction of the periprosthetic blood flow to the abdominal branches adjacent to the celiac trunk with a substantial interruption of the blood circulation at the trunk itself was assessed. However, collateral circles supplied the corresponding areas.

Moreover, the patient had a history of chronic obstructive pulmonary disease (COPD), dyslipidemia, hypertension, and clinically stable hypertensive heart disease with a preserved ejection fraction and good circulatory compensation.

### Macroscopical and histological findings

**Fig. 3:**
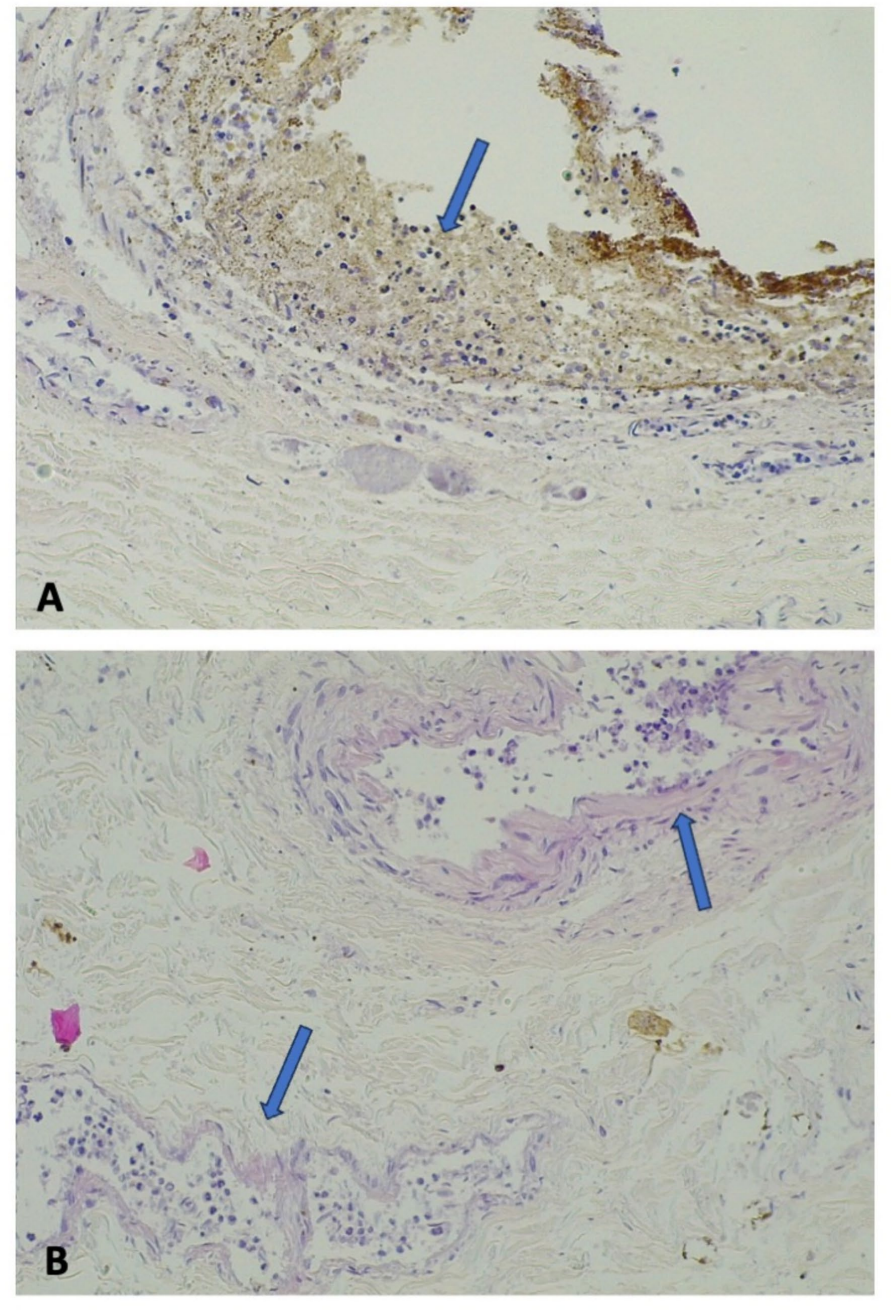
Gastric wall: the autolysis was severe, but neutrophils were present within the mucosa (**a**, hematoxylin and eosin 20HPF) and in the lumen of the parietal vessels (**b**, hematoxylin and eosin 20HPF)

After fixation in formalin, the SMA was serially sectioned and showed no signs of blood clotting. When examined with hematoxylin and eosin staining, the gastric wall appeared autolytic, but residual signs of infarction were detected, such as the presence of neutrophils in the mucosa and leukocytosis in the small parietal vessel (Fig. 3).

**Fig. 4:**
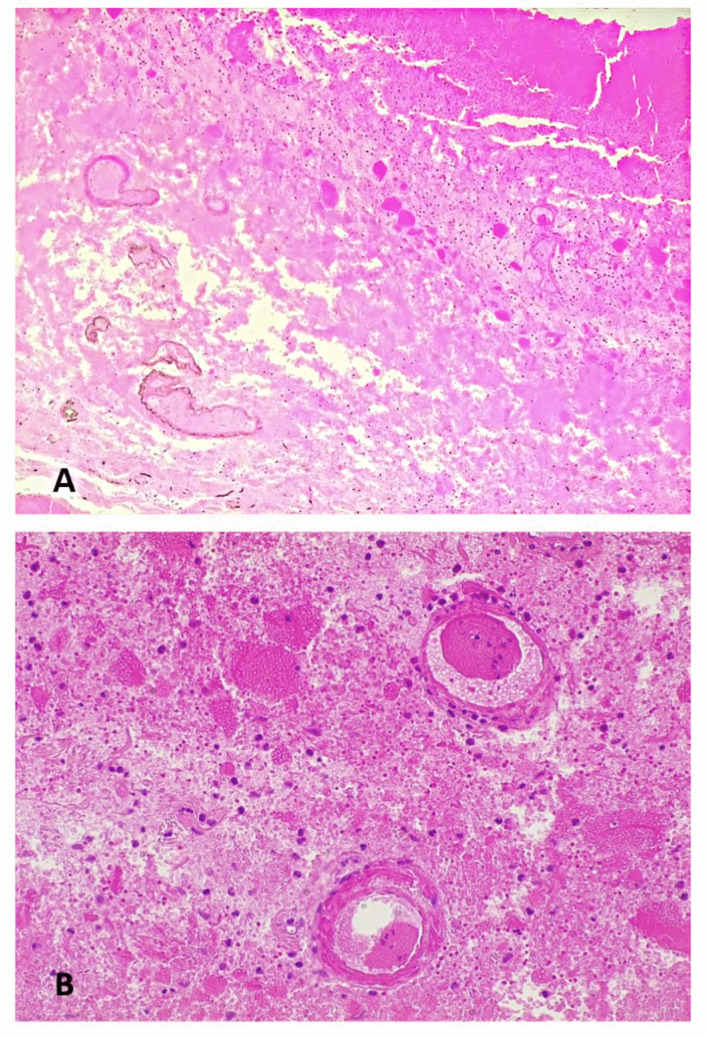
Ischemia/infarct of the duodenojejunal flexure: the intestinal wall was overall autolytic, but there was diffuse erythrocyte extravasation (**a**, hematoxylin and eosin 4HPF) and small non-occlusive thrombi within the vessels of the submucosa were noted (**b**, hematoxylin and eosin 20HPF)

**Fig. 5:**
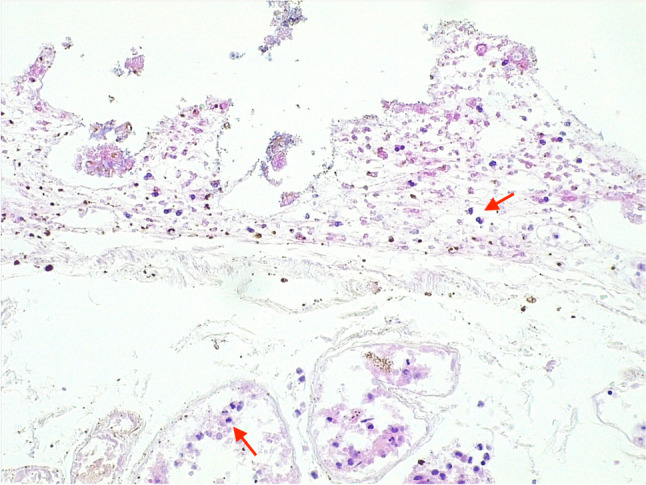
Jejunal ischemia/infarct: the intestinal wall showed severe autolysis, but neutrophils were still recognizable within the mucosa (upper arrow) and the capillaries of the submucosa (lower arrow) (hematoxylin and eosin 20HPF)

The duodenojejunal flexure and the first part of the jejunum also displayed widespread autolysis, but histological features indicating ischemia or hemorrhagic infarction were still evident. The duodenojejunal flexure presented diffuse erythrocyte extravasation within the wall and small non-occlusive thrombi in the small vessels of the submucosa (Fig. 4). Although post-mortem changes were prominent in the first part of the jejunum, neutrophils within the mucosa and the submucosal vessels could still be identified (Fig. 5).

**Fig. 6:**
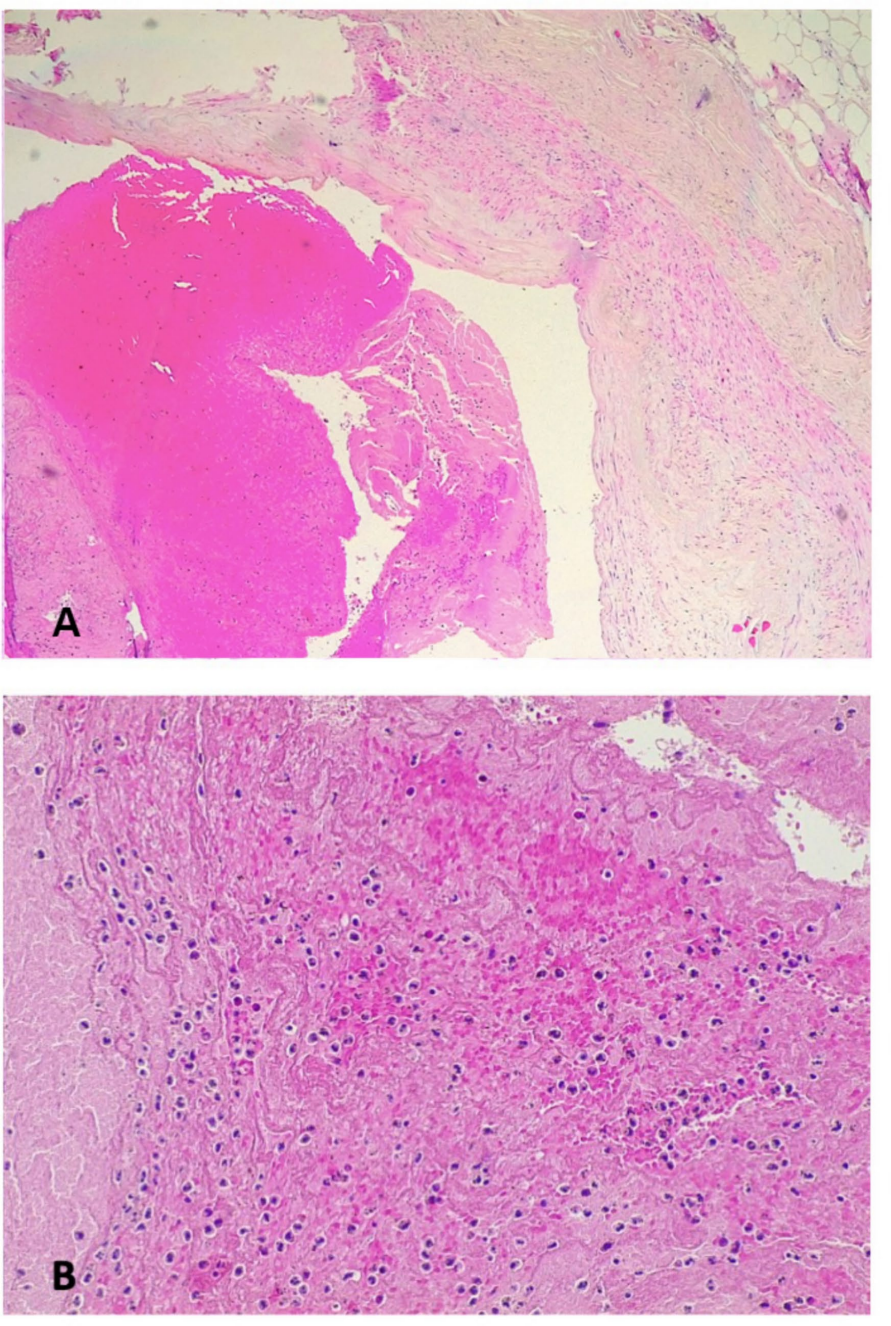
Superior mesenteric artery: the lumen presented focal non-occlusive initial thrombosis (**a**, hematoxylin and eosin 4HPF) in which fibrin strands and neutrophils were recognizable (**b**, hematoxylin and eosin 20HPF)

The SMA presented non-occlusive, focal and initial thrombosis characterized by fibrin strands and neutrophils (Fig. 6).

The examination of the heart revealed some foci of interstitial fibrosis, but no evidence of acute ischemia, necrosis or lymphocytic infiltration was found. The liver did not show any signs of fat accumulation. Lungs exhibited signs of putrefaction, as well as pulmonary vascular congestion and edema. Acute tubular necrosis was shown in the kidneys, but no signs of cortico-medullary junction necrosis were detected.

## Discussion

We present here a case of gastric and bowel ischemia presenting to the forensic facilities without a prior diagnosis before the unexpected death.

Gross examination revealed a blackish and sharply discolored stomach and first tract of the jejunum, that oriented towards ischemia and infarction. Nevertheless, given the typically redundant blood flow to the stomach [9] initial consideration was given to putrefaction changes as the cause of discoloration, since they are known to act as confounding factors for the diagnosis of bowel ischemia [3]. Despite the relatively long post-mortem interval of our case, other distal intestinal loops appeared regularly pinkish, so a significant influence of post-mortem decay was ruled out.

Histologically, despite marked autolytic changes, signs of gastric and intestinal ischemia/infarction were still detected, including the presence of neutrophils in the mucosa and the small parietal vessels. The duodenojejunal flexure showed diffuse erythrocyte extravasation and small non-occlusive thrombi within the submucosal vessels. Both macroscopic and microscopic findings were thus consistent with gastrointestinal ischemia/infarction.

The symptoms and signs exhibited by the patient, including abdominal pain and distension, vomiting, diarrhea and anemia, also corresponded to the clinical picture of ischemia affecting the stomach or bowel, albeit non-specific [9, 11, 27].

Regarding the cause of gastric and bowel ischemia, as already stated, bowel ischemia is commonly classified as occlusive mesenteric and non-occlusive [28]. In our case, the autopsy revealed an occlusion of the celiac trunk, which is a plausible cause of ischemia [29]. However, the clinical data reviewed for the investigation proved fundamental in assessing that this blockage to the blood flow had occurred several years prior and could not therefore be responsible for the acute ischemia.

Although multiple CTs showed revascularization of the stomach by collateral vessels, the reviewed health care data also revealed an unusual and rather precarious vascularization pattern of the stomach. In this peculiar context of collateral revascularization due to occlusion of the celiac trunk, the SMA might have provided a partial blood supply to the stomach. However, the occlusion of the SMA would have resulted in a more extensive necrosis, also affecting other vascularized intestinal tracts, such as the small intestine, the right and transverse large bowel [30]. Instead, the ischemia/infarction was limited to the stomach and the first tract of the small bowel.

The non-occlusive type of bowel ischemia seemed more plausible also due to the fact that the SMA was not completely occluded, showing focal areas fibrin strands and neutrophils.

In the non-occlusive AMI, intestinal necrosis is caused by local vasoconstriction in a condition of abdominal hypovolemia, reduced cardiac function, or vasoconstrictor medications [28]. Gastric ischemia has also been associated with non-occlusive causes, such as systemic hypoperfusion in shock, sepsis or severe atherosclerosis [8–10, 27, 31]. However, many cases remain idiopathic [27].

In the case here presented, the patient displayed compromised circulation in the usually rich vascular network of the stomach and this likely predisposed her to transient reductions in splanchnic circulation. She also presented advanced age, severe atherosclerosis, and hypertension, which are reported in the literature as risk factors for gastric ischemia [32].

No other causes of systemic hypoperfusion were identified, such as third space losses and systemic inflammatory response syndrome (SIRS) or SIRS-multiple organ dysfunction syndrome (MODS). Particularly, no diffuse alveolar damage, histopathological markers of acute respiratory distress syndrome (ARDS), reduced coronary perfusion or increased inflammatory mediators in the organs were found [33]. However, microbiological analyses were not performed.

The acute tubular necrosis, cortical thinning and blurred cortico-medullary junction were considered indicative of chronic kidney disease, given the patient’s history and age. Nevertheless, the pre-existing conditions, including COPD, heart and kidney failure, likely compromised the woman’s ability to withstand the acute gastric ischemia.

The multiple episodes of vomit and diarrhea might have exacerbated dehydration and hypovolemia and might have led to further hypoperfusion. The disruption of the delicate balance of circulatory compensation might have resulted in gastro-intestinal ischemia/infarct.

Since no mechanical obstruction, perforation, or peritonitis were found, and occlusive thrombosis of the SMA was absent, the ischemia was classified as non-occlusive and a likely result of a systemic disequilibrium.

Intestinal ischemia carries a high mortality rate of 60–80%, as the alteration of the mucosal barrier triggers cytokine and reactive oxygen species (ROS) release. These molecules cause damage to the microcirculation and facilitate bacterial migration, leading to subsequent reperfusion injuries due to neutrophil chemotaxis and ROS production [28]. Gastric ischemia has an estimated mortality rate of 30–40% [27].

Given the rarity of the condition, the non-specific symptoms, the compromised health status of the patient, and the very high mortality rate, no medical liability could be proven with scientific evidence. On the other hand, the case here presented highlights that gastric ischemia could be encountered both in the clinical scenario, where the level of suspicion should be very high, and in the post-mortem context.

Given that the autopsy might remain the only opportunity to achieve a diagnosis, it is strongly suggested to gather comprehensive medical history data to avoid misinterpretations, and to document all relevant associated comorbidities.

The forensic pathologist should consider the possibility of gastric ischemia, particularly when examining older people with a history of hypertension, atherosclerosis, previous aortic surgery, and impairment of the gastric supply, as well as other causes for hypovolemia. This is especially crucial for older women, who are estimated to spend almost 12 years in ill health [5].

Considering the very high prevalence of cardiovascular diseases in the aging population, especially among women, and the variations in morbidity and mortality by age in older women, forensic pathologists should be aware of the possibility of encountering previously uncommon conditions, as in the case presented.

## Conclusions

The reported case suggests the need for a comprehensive evaluation of forensic evidence including the analysis of clinical data especially when a rare cause of death is suspected. Considering the expected consistent increase in life expectancy, especially for women, and the rise in morbidity, especially of vascular nature, it's important to focus attention on both the clinical and the medicolegal diagnosis of gastric ischemia.

## Key points

1. Gastric ischemia is a rare cause of death due to the rich vascular supply and it has some confounding factors, including putrefaction.
2. Gastric ischemia could give rise to suspicions of medical liability.
3. The analysis of clinical data and the documentation of comorbidities could be fundamental to highlight risk factors for non-occlusive gastric ischemia.
4. Due to variations in morbidity and mortality by age, especially in older women, gastric ischemia could be increasingly encountered by forensic pathologists.
